# A Comprehensive Survey of Retracted Articles from the Scholarly Literature

**DOI:** 10.1371/journal.pone.0044118

**Published:** 2012-10-24

**Authors:** Michael L. Grieneisen, Minghua Zhang

**Affiliations:** 1 Wenzhou Medical College, Wenzhou, Zhejiang, China; 2 Department of Land, Air and Water Resources, University of California Davis, Davis, California, United States of America; IUMSP, University Hospital Lausanne, Switzerland

## Abstract

**Background:**

The number of retracted scholarly articles has risen precipitously in recent years. Past surveys of the retracted literature each limited their scope to articles in PubMed, though many retracted articles are not indexed in PubMed. To understand the scope and characteristics of retracted articles across the full spectrum of scholarly disciplines, we surveyed 42 of the largest bibliographic databases for major scholarly fields and publisher websites to identify retracted articles. This study examines various trends among them.

**Results:**

We found, 4,449 scholarly publications retracted from 1928–2011. Unlike Math, Physics, Engineering and Social Sciences, the percentages of retractions in Medicine, Life Science and Chemistry exceeded their percentages among Web of Science (WoS) records. Retractions due to alleged publishing misconduct (47%) outnumbered those due to alleged research misconduct (20%) or questionable data/interpretations (42%). This total exceeds 100% since multiple justifications were listed in some retraction notices. Retraction/WoS record ratios vary among author affiliation countries. Though widespread, only miniscule percentages of publications for individual years, countries, journals, or disciplines have been retracted. Fifteen prolific individuals accounted for more than half of all retractions due to alleged research misconduct, and strongly influenced all retraction characteristics. The number of articles retracted per year increased by a factor of 19.06 from 2001 to 2010, though excluding repeat offenders and adjusting for growth of the published literature decreases it to a factor of 11.36.

**Conclusions:**

Retracted articles occur across the full spectrum of scholarly disciplines. Most retracted articles do not contain flawed data; and the authors of most retracted articles have not been accused of research misconduct. Despite recent increases, the proportion of published scholarly literature affected by retraction remains very small. Articles and editorials discussing retractions, or their relation to research integrity, should always consider individual cases in these broad contexts. However, better mechanisms are still needed for raising researchers’ awareness of the retracted literature in their field.

## Introduction

The number of articles retracted each year has increased precipitously in recent years. Prior studies, which mainly focused on the medical literature, found that article retractions and lapses in research integrity impact both the published literature and the evolution of scientific knowledge [1]–[20]. However, the severity of the problem has been a matter of debate. On one hand, some feel that the rise in retractions reflects a very real and pressing issue: Although retracted publications represent only a miniscule percentage of the total literature [1], [2], surveys of researchers have suggested that only a fraction of research misconduct cases are caught and publicly discredited [3], [4]. Furthermore, the results from retracted articles continue to be cited as valid [5]–[7]. In an attempt to determine the potential extent of the problem, a crude estimate of “potentially retractable” articles based on high-impact journal retraction frequencies was conducted [1], though it has since been criticized as too simplistic [8].

In contrast, other authors argue that since articles can be retracted for a variety of reasons, the recent rise in retractions may not actually reflect a “crisis of scientific integrity” which may be superficially suggested by the raw numbers: For example, past surveys found that despite an increasing number of retractions due to misconduct [9], [10], more articles had been retracted due to unintentional errors [11]. For this reason, some have argued that article retraction should generally be uncoupled from the stigma of “misconduct” [12]. They argue that if retractions are to be used as a proxy for measuring misconduct, then retraction, or “unpublication,” should be a last resort, reserved for only the most egregious offences [13], [14].

Recent systematic studies have attempted to characterize retraction notices, retracted publications and the role of misconduct in order to gain a more thorough understanding of the impact of retractions and the true extent of misconduct in scholarly research. However, studies published from 1992–2011 were somewhat limited in scope due to a sole reliance on the literature indexed in the PubMed database (Table S1), with the largest dataset examined to date comprising only 871 retracted publications [9].

In order to gain a broader perspective on the phenomenon of article retractions, in both the medical and non-medical literature, we identified 4,449 formally retracted scholarly publications from 42 bibliographic databases and major publisher websites representing a broad spectrum of scholarly fields. Our analysis investigated various attributes among these articles, such as their distributions across disciplines, geographic location of author affiliations, the justifications and authorities calling for retraction, and temporal trends. Trends in these attributes can shed light on why publications are being retracted at current rates, in both the medical and non-medical literature, and foster the development of more effective methods for curbing the rising retraction rates.

## Materials and Methods

### Data Sources

Retracted scholarly articles were identified using bibliographic databases, major publisher websites, non-publisher journal aggregators (such as J-STOR), and search engines. To compile a comprehensive list of retracted articles, a wide variety of data sources must be consulted for several reasons. Bibliographic databases vary with respect to the journals they index “cover-to-cover,” the journals they index only selectively, and their policies regarding retroactively marking existing records for retracted publications [21], [22]. Due to the latter two factors, even if database X covers journal Y, identifying the retracted articles from that journal in the database may not be possible. One example is the Inspec database, which covers a wide range of engineering journals but does not index retraction notices or retroactively mark retracted article records in any discernible way.

Thus, 42 data sources were consulted from May-June 2011 using the queries indicated in Table 1. The highest-yielding sources were re-queried in Aug 2011 to capture the dozens of articles retracted in the interim. Search terms for retrieving either the retracted articles or the retraction notices were used, as cases where only one of the two could be identified in a given data source were common. The 42 data sources consulted represent the largest broad-scope scholarly literature databases, the most comprehensive sources which focus on major fields of study, and several which cover specialized literature that is largely excluded from other sources. An example of the latter is *Global Index Medicus-IMSEAR (SEARO)*, which yielded many retracted articles from Southeast Asian medical journals that are not indexed in any of the other sources.

**Table 1:** Forty-two data sources were consulted to locate retracted articles.

A. Multi-publisher databases and journal aggregators:
**1. Academic Search Complete** – TI = retract* (n = 1944 on 2011.07.15)
**2. Bioone** – TI = retract* (n = 10 on 2011.08.26)
**3. Biosis** – **[A]** (AU = (A* to Z*) and PT set to “Retraction”) [Note: It is not possible to directly search Biosis by the PT field.]**;** **[B]** (TI = retract* NOT [A]) (n = 4265 on 2011.08.07); **[C]** TI = (withdraw* and (article* or publication* or paper* or manuscript*)) (n = 26 on 2011.08.05)
**4. CAB Abstracts** – TI = retract* (n = 158 on 2011.08.07)
**5. CiNii** – TI = retract* (n = 699 on 2011.08.07)
**6. COMPENDEX** – TI = retract* (n = 847 2011.08.07); withdraw* (none referred to “withdrawn articles”)
**7. CSA Illumina – Natural Sciences** – TI = retract* (n = 1164 on 2011.07.15)
**8. CSA Illumina – Social Sciences** – TI = retract* (n = 297 on 2011.07.15)
**9. CSA Illumina** **– Technology** – TI = retract* (n = 164 on 2011.07.15)
**10. DOAJ** – **[A**] TI = retraction (73 on 2011.08.22); **[B]** TI = retracted (10 on 2011.08.22); **[C]** TI = withdrawn (none referred to “withdrawn articles”)
**11. EconLit** – TI = retract* (n = 8 on 2011.05.21)
**12. Federal Register** – misconduct AND (findings or research or scientific or science)
**13. Global Index Medicus-IMEMR (EMRO)** – TI = retract, retracted, retraction (n = 15 on 2011.08.26)
**14. Global Index Medicus-IMSEAR (SEARO)** – TI = retract* (n = 36 on 2011.05.16)
**15. Global Index Medicus-WPRIM (WPRO)** – **[A]** TI = retract(ed)(ion) (n = 155 on 2011.08.26); **[B]** TI = withdraw(al)(n) – none relevant
**16. Global Index Medicus-LILACS** – TI = retract* or retracc* or retração (n = 122 on 2011.05.16)
**17. Inspec** – (retract*) yielded 143 hits (2011.08.22) (none referred to “retracted articles”)
**18. J-STAGE** – TI = retract* (n = 70 on 2011.08.22)
**19. JSTOR** – TI = retract* (n = 457 on 2011.08.07)
**20. MathSciNet** – TI = retract* (n = 1046 on 2011.08.05)
**21. MUSE** – TI-retract* (n = 4 on 2011.08.26)
**22. PubMed** – **[A]** “retracted publication”[pt] or “retraction of publication”[pt] or “retraction”[ti] or “retractions”[ti] or “retracted”[ti] (n = 6736 on 2011.08.22)**;** **[B]** withdrawn[ti] (n = 1275 on 2011.08.26); **[C]** withdrawal[ti] - many hits but none were relevant
**23. SciELO** – retraction/retracción/retração – none referred to retracted articles
**24. Wanfang (Chinese jrnls)** – TI = retract* (n = 193 on 2011.05.12)
**25. Wanfang (English jrnls)** – TI = retract* (n = 5 on 2011.05.12)
**26. Web of Science (WoS)** – **[A]** TI = retract* (n = 7925 on 2011.08.07); **[B]** TI = (withdraw* and (article* or paper* or publication* or manuscript*)) NOT (TI = retract* OR SO = “Cochrane Database of Systematic Reviews”) (n = 41 on 2011.05.28)
**B. Search engines**
**27. Google Scholar** – “retracted at the request” “article has been retracted” “article has been withdrawn” (n = several hundred hits for each phrase, 2011.07.15)
**28. Scirus** – TI = retract* (n>35,000, only consulted individual “journal sources” subsets not previously consulted)
**C. Publisher websites and individual journals (all searched 2011.08.26):**
**29. American Society of Microbiologists** – TI = retract* (n = 20)
**30. American Chemical Society** –KW = retract* and limit to “corrections”
**31. Blood** – TI = retract or retraction or retracted (n = 23)
**32. Emerald** – TI = retract* (n = 14)
**33. FASEB Journal** – TI = retract* (n = 17)
**34. IEEEXplore** – [no field specified] (violation and ieee and principles) = 374; retracting = 218; retracted = 214; withdrawn = 443 (all 2011.08.05)
**35. InformaHealthCare** – TI = retract* or withdraw* (n = ∼400)
**36. IOS Press** – TI = retract*
**37. Liebert** – TI = retract* (n = 27)
**38. PNAS** – TI = retract* (n = 40)
**39. Sage journals – TI = **“retract*” (n = 98)
**40. ScienceDirect** – **[A]** TI = retraction or retracted (n = 1740); **[B]** TI = errat* and KW = retract* (n = 61)
**41. Springer** – TI = retracted, retraction, retractions) (n = 586)
**42. Wiley** – TI = retract, retracted, retraction (n = 684 on 2011.08.23)

For each source, the specific query used and number of records returned during the most recent query are indicated. Field abbreviations are: KW = key word; PT = publication type; SO = source (i.e. journal) title; TI = title.

### Criteria for Considering an Article “retracted”

Here we consider a “retracted” article to be one that has been explicitly “retracted” or “withdrawn” via a notice, erratum, corrigendum, editorial note, “rectification,” or other such editorial notification vehicle. We included cases of “partial retraction” by such notices, where retraction applied to only a portion of the publication, such as a single figure with questionable data which may or may not be central to the main point(s) of the publication. Articles identified as problematic but not explicitly retracted (such as a simple “statement of duplicate publication,” pairs of original and “corrected and republished” articles, or those mentioned in “editorial expression of concern” notices (but not yet retracted) were not included in this study.

### Compiling the Master list of Retracted Articles

Compiling the list of retracted articles and their corresponding retraction notices began with the PubMed query (“retracted publication”[pt] or “retraction of publication”[pt]). Each retracted article citation in the results output was matched up with the citation for its corresponding retraction notice based on either data from the PubMed records or consultation of the notice to determine which article(s) it retracted. The list of paired retracted article/notice citations was imported into an Excel spreadsheet and sorted by full journal title, volume, and pagination of the original article. The results of additional queries of PubMed and other data sources listed in Table 1 were sorted by “journal title” (for databases offering such an option) and all results were manually screened against the growing retracted article list. Duplicates and “off-topic” hits were discarded, while citations for clearly identified retracted articles and retraction notices that were not already on the list were added. Terms such as “retraction” and “withdrawal” are used in many contexts other than article retractions, such as “retraction” of an airplane’s landing gear or “alcohol withdrawal.” Thus, many queries yielded high proportions of “off-topic” hits–such as the WoS query for “TI = retract*” which yielded 7,925 records on 07 Aug 2011 (Table 1, line 26). Additional data sources were queried until the vast majority of retracted articles retrieved were already on the list, indicating a point of diminishing returns. At that point, queries of the websites of individual journals with large numbers of retractions (Table 1, part C) verified that we had identified all retracted articles in them from the data sources consulted. In summary, the 42 data sources yielded 4,449 scholarly articles retracted between 1928 and 2011, which were subjected to further analysis.

### Metadata

In order to characterize various attributes of the retracted articles, we were able to obtain 4,244 of the retracted articles and the retraction notices for 4,232 of them. In the remaining cases, the article, its retraction notice, or both were not available to us in hard copy or online. Because our survey yielded only 21 articles retracted prior to 1980 and 2011 was only a partial year, our analysis focused on the retracted articles published from 1980–2010. Previous smaller-scale studies have examined various attributes of retracted articles from the medical literature (Table S1), such as trends in number of articles retracted over time, across disciplinary fields, and by country of authorship; in addition to determining the frequency of various justifications for retraction and the authorities involved in calling for the retraction of articles. We sought to examine these attributes among a more comprehensive and multi-disciplinary set of retracted articles. Table 2 lists the categories used to describe the attributes of the retracted articles that we analyzed in this study. The information given in retraction notices was taken at face value, and no attempt was made to independently verify the accuracy of the statements made in the notices.

**Table 2:** Attributes of article retraction cases analyzed in this study.

**1. Justification for retraction:** Many retraction notices list multiple retraction justifications for a single article. For the purposes of this study, the justifications were divided into categories of Publisher error, Author error, Other and Unspecified. These broad categories were then further divided, resulting in a total of 15 justification categories:
**1.a. Publisher error**
1.a.i. Accidental duplicate publication
1.a.ii. Preliminary version accidentally published (often “version without final author corrections”)
1.a.iii. Published in wrong journal
1.a.iv. Special issue, when article was accidentally published in either a special or regular issue, though intended for the other
1.a.v. Other – including cases where an article was rejected but subsequently published in error
**1.b. Author error**
1.b.i. Research misconduct
1.b.i.1. Allegations of data fraud, including data falsification, fabrication or manipulation, or intentionally biasing research design to favor a particular outcome
1.b.i.2. Other, such as failure to obtain legally required oversight for conducting the research, usually institutional review board approval of medical research
1.b.ii. Publishing misconduct
1.b.ii.1. Plagiarism of either text or figures from works of others, when no common authors exist between the two publications
1.b.ii.2. Author-initiated multiple publication (“duplicate publication”) where the separate submissions or publications have at least one author in common; cases of “self-plagiarism” or re-publishing one’s own data previously published elsewhere, without acknowledgment of the original publication or permission from the copyright holder are included here
1.b.ii.3. Authorship issues – mainly failure to consult or inform all listed authors about the submission of the publication, or excluding authors who contributed substantially to the work
1.b.ii.4. Vague “copyright issues” or “legal concerns” stated in the absence of sufficient detail to assign to one of the 3 previous categories
1.b.ii.5. Other
1.b.iii. Distrust data or interpretations, meaning that the data or interpretations as published are no longer considered valid or reliable by some or all of the authors. This category is dominated by cases of unexplained data irreproducibility or experimental artifacts discovered post-publication, and excludes cases of “data falsification or fabrication” covered by category 1.b.i.1 above.
**1.c. Other** – including scenarios where results from a crucial support article were retracted, or statements such as “breach of ethics” or “data irregularities” which were too vague to allow proper assignment to any of the more specific categories.
**1.d. Unspecified** – A sizeable percentage (∼18%) of notices gave no reason justifying the retraction
**2. Retraction authorities:** Retraction authorities specifically mentioned in the retraction notices were classified under one of the following categories:
2.a. Publisher
2.b. Editor(s)
2.c. Some authors
2.d. All authors
2.e. Lawyer (or legal counsel)
2.f. ORI for Office of Research Integrity (Department of Health and Human Services USA)
2.g. Institute, when institutional investigations are mentioned
2.h. Other; or
2.i. Not specified
**3. Scholarly fields.** The retracted publications were assigned to scholarly fields based on Web of Science (WoS) categories assigned to the journals in which they are published. The number of articles in each WoS category were then tallied. These figures were then summed by assigning WoS categories to one or more of 12 broad fields in Fig. 2. In addition, the impact factor of each journal listed in the 2010 edition of Thompson Reuters’ Journal Citation Reports was obtained.
**4. Country of affiliation.** Defunct country names were combined as appropriate to reflect current United Nations-recognized countries. The European Union (EU-27) category included retracted articles with at least one author from one of the 27 countries comprising the EU as of 2011. In some cases where original articles were not available, author affiliation countries were obtained from WoS, the only major database which includes all author affiliation addresses. In total, there were 102 countries represented.
**5. Year.** Articles were categorized by the year of publication and the year of retraction. For articles retracted prior to volume/pagination assignment, the date posted online was used, when known.
**6. Full or partial retraction.** Some notices only retracted a portion of a publication, such as a single figure with questionable data which may or may not be central to the main point(s). These were considered ‘partial’ retractions, to distinguish them from full retractions. These “partial retractions” represented 3% of the articles included in our analyses.

### Retraction “rates”

While raw counts of retracted articles are appropriate for comparing certain attributes, for others adjusting figures for the different sizes of subcategories being compared is more appropriate. This allows us to think in terms of “how prevalent are retracted articles”–1 in 1,000?, 1 in 1,000,000?–in different disciplines, for example, which differ in the size of the published literature. For comparisons between different years, disciplines, journals, and authorship countries, data are presented as both raw counts and either ratios or percentages which are normalized by some measure of their relative sizes.

### Attributes Studied

#### Disciplinary and journal distributions

An objective system for assigning articles to various fields of study is required to compare retraction rates among different scholarly disciplines. WoS covers all scholarly fields and includes query features for easily obtaining data on the relative proportions of articles published in specific disciplines. The retracted publications we identified were assigned to scholarly fields based on the “Web of Science Categories” assigned to the journals in which they are published. Only those journals indexed by WoS were assigned WoS Categories. The remaining journals were not indexed by WoS so the retracted articles in them were excluded from those analyses which rely on additional WoS data. For each of the WoS Categories with at least one retracted article, the sum of retracted articles in the journals assigned to that category gives the **number** of retracted articles in the category.

However, the size of the published literature varies among fields of study; therefore, the raw counts must be normalized to allow direct comparison of the **proportions** of articles which are retracted in each field. Percentages of records in each WoS Category for a WoS query of “publication year = 2010” were obtained using the “Web of Science Categories” option of the “Analyze Results” feature of WoS. These percentages were then compared with the percentages of the retracted articles which were assigned to these same categories. The WoS Categories were also grouped into 12 broad fields. The retracted articles in each WoS Category were summed and the sums were converted to percentages of the retracted articles. Results from the WoS query of “publication year = 2010” were grouped into the same 12 broad fields, and converted to percentages of all records for each field. Because previous studies of retracted articles focused on the literature indexed in PubMed, we also compared the number of “PubMed retractions” to “non-PubMed retractions” for each year from 1980–2010; with “PubMed retractions” defined as those articles that are marked with “Retracted publication” in the publication type field.

#### Justification for retraction

Various justifications have motivated the decisions to retract scholarly articles. Determining the relative proportions of these justifications shows their impact on the published literature. Many surveys of the retracted literature (see references in Table S1) use justifications categories dictated by the focus of the study. For example, a study of “scientific fraud” [10] divided all justifications into “Fraud” and “Error” categories, and included breaches of publishing ethics (*e.g.*, plagiarism, duplicate publication, authorship disputes) in the latter group. In this study, we separately quantified several overlapping justification categories (Table 2) which are relevant to different issues. For example, the category total for “Alleged data manipulation” shows the scope of this particular issue. On the other hand, determining the overall extent of unreliable published data requires a count of all articles with data questioned due to either “Alleged data manipulation” or “Distrust data or interpretations” (the latter including artifacts or unexplained irreproducibility). Because multiple justifications are often given for retraction of a single article, the counts for individual categories can not simply be added together.

From our initial list of justification categories, some were refined as logical groupings emerged among the retraction notices consulted, and all notices previously assigned to a category which changed were reassessed. For example, we initially included separate categories for “known artifact” and “unexplained irreproducibility,” but we decided to merge them into one category of “Distrust data or interpretations” after we noticed varying degrees of certainty expressed in different cases. None of the retraction notices in this study specifically mentioned ghostwriting or guestwriting, activities receiving increasing attention in recent years [23].

#### Repeat offenders

While compiling the list, we noticed that large numbers of retracted articles were sometimes associated with a single author. Some of these cases involved an extensive list of publications in a single retraction notice, while in others, retraction notices were scattered among the individual journals involved. To determine the influence of specific individuals on total retraction counts, the author lists for all 4,449 retracted articles were subjected to a Pivot Table analysis in Excel. Author names yielding more than 15 hits were then surveyed to determine how many articles were attributable to a single individual based on field of study and institutional affiliation.

#### Retraction rates per year and “publication inflation”

Increasing numbers of article retractions per year have been noted in recent studies of the PubMed literature [1], [2], [5], [9]–[11], [19], [24]–[30]. Here, retracted articles in all disciplines were summed for each year from 1980–2010 based on the year that the retraction notice was published. However, the size of the literature published each year has also increased (“publication inflation”) during this period. To gauge how much of the increase in numbers of retractions per year is simply due to overall growth of the published literature, changes in the number of database records in PubMed and WoS for each year were used as proxies for “publication inflation”. The total numbers of WoS records for each year were obtained using the “Publication Years” option of the “Refine Results” feature of WoS for a query of “publication year = 1980–2010”. Individual queries of PubMed for each year using the “[dp]” field tag (*e.g.*, “2010[dp]”) yielded the number of records for each year. Raw numbers of articles retracted each year were then expressed as percentages of PubMed and WoS records for the same year.

However, some articles are retracted many years (or even decades) after publication. Since WoS added 14.48 million records from 2001 to 2010, the pool of articles which could potentially be retracted was much larger in 2010 than it was in 2001. These two factors led us to wonder if it would be more appropriate to consider rising retraction rates in the context of the size of the body of literature from which most retractions are drawn in a given year, rather than the size of the literature published in the year of retraction. Years of both publication and retraction were known for 3,545 of the articles retracted from 1980–2010. Of these, 78.6% were published in either the year of retraction or the 3 preceeding years–*e.g.*, 478/591 of the articles retracted in 2010 were published during the 4-year period of 2007–2010 (Table S2). Therefore, the growth of sequential 4-year sums of WoS record counts can represent the growth of the pool of articles which includes most of the articles retracted in a given year. The change in retraction rate, adjusted for this factor, is considered by comparing the change in the two ratios:
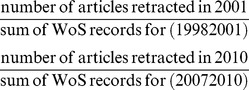



#### Geographic distribution of authors

Prior studies have discussed geographic trends in the authorship of retracted articles [15], [20]. Since WoS lists all author affiliation addresses (rather than just the author of correspondence) we were able to obtain the countries of all author affiliations for 4,244 retracted articles. Pivot Table analysis in Excel yielded counts for each of the 101 countries. To determine any changes in geographic distribution over the years, counts for individual retraction years from 1980–2010 were obtained for the geographic entities with highest counts (USA, EU27, China, India, Japan and South Korea) using Excel Pivot Tables. Because the scientific output of these regions varies, raw counts were then divided by the number of WoS records for each country-year combination. These figures were obtained by querying WoS for individual years (*e.g.*, “PY = 2010”) and using the “Countries/Territories” option of the “Analyze Results” feature on the results for each year. The resulting ratios approximate the **relative** proportions of published articles for each country which have been retracted. However, since WoS does not index the total scholarly output of any country, these should not be considered absolute ratios of retracted articles.

#### Retraction authorities

To better understand the roles of various authorities in the retraction of articles, for each case where the retraction notice was obtained the authorities specificially mentioned in the notice were recorded based on the categories in Table 2. We obtained the retraction notices for 4,232 articles (95.1% of 4,449 total), and specific authorities were mentioned in the notices for 3,510 retracted articles (82.9% of 4,232).

## Results

### Journal Distribution

Retractions were identified in 1,796 unique journal titles, including 59 (64%) of the 92 research journals with a 2010 ISI Impact Factor of 9.000 or higher (Table S3). Journals ranged from 1–128 retractions over the period of Jan 1980 to Sep 2011. Only 22 journals (1.2%) had over 15 retractions, and the percentages of articles published since 1980 which were retracted by 22 Sep 2011 ranged from 0.02–5.62% (Table 3). The footnotes show the dramatic effects that individual authors or editorial errors can have on these rates. Among the remaining journals only 0.02–0.16% of articles published since 1980 were retracted.

**Table 3:** Twenty-two journals each had 15 or more retracted articles.

Journal title abbreviation	Number of retracted articles	WoS records since 1980	Percent of articles retracted
*Acta Crystallogr E*	123	31,152	0.39^1^
*Science*	73	76,801	0.09
*PNAS*	73	85,064	0.08
*J Biol Chem*	59	130,667	0.04
*Gene Expr Patterns*	49	871	5.62^2^
*Nature*	47	97,384	0.05
*Anesth Analg*	40	23,632	0.17^1^
*Biochem Biophys Res Commun*	36	57,026	0.06
*J Immunol*	33	50,451	0.06
*Blood*	29	123,171	0.02
*J Hazard Mater*	25	10,678	0.16^3^
*J Am Chem Soc*	24	76,644	0.03
*Cell*	23	14,718	0.16
*J Clin Invest*	22	16,830	0.13
*Tissue Eng Regen Med*	20	532	3.76^4^
*N Engl J Med*	18	54,555	0.04
*Hear Res*	16	5,362	0.30^5^
*Appl Phys Lett*	15	83,838	0.02
*EMBO J*	15	16,060	0.09
*FEBS Lett*	15	38,101	0.04
*Infect Immun*	15	24,306	0.06
*Mol Cell Biol*	15	20,466	0.07

1 Two authors, H. Zhong and T. Liu, accounted for most of the retractions from *Acta Crystallogr E*, as did Joachim Boldt for *Anesth Analg*.

2 All 49 articles from *Gene Express Pattern* were retracted due to a publisher error in which an entire issue of journal *Mech Dev* was accidentally published as *Gene Expr Patterns*. This journal was not covered in its entirety in Web of Science, so the count of 871 “records since 1980” is from PubMed.

3 17 of 25 *J Hazard Mater* retractions were authored by Pattium Chiranjeevi.

4 The count of 532 articles since 1980 is an underestimate since WoS only includes articles for volume 4 onward for this journal, and it is not covered by PubMed. This is a Korean-language journal to which we have no access, so the reason for this large percentage is not known.

5 12 of the 16 retractions for *Hear Res* were articles accidentally posted online on two different dates (2009 Oct 8 and 2010 Jan 30), indicating two isolated editorial errors.

### Disciplinary Distributions

**Figure 1:**
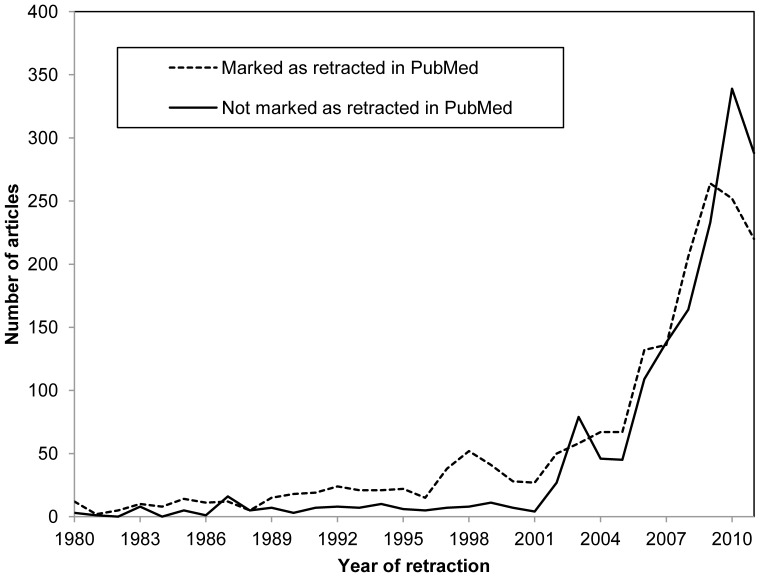
Scope of “non-PubMed” retracted articles. Of 3,490 retracted articles with a known year of retraction, 1,880 were labeled with “retracted publication” in the publication type field in the PubMed record (on 21 Sep 2011), and 1,610 were not. The prominence of retracted articles outside of PubMed has increased since 2002.

The number of “PubMed retractions” per year outnumbered non-PubMed retractions until 2002, indicating the dominance of the medical literature among retractions in the past (Fig. 1). However, roughly equal numbers have appeared each year thereafter, with 1,402 PubMed and 1,442 non-PubMed retractions appearing from the beginning of 2003 to 22 Sep 2011.

WoS Category assignments were available for 1,522 (85%) of the 1,796 different journals which included at least one retracted article. From a total of 244 possible WoS Categories, retracted articles were found in 201 of them (Fig. S1). Among these 201 WoS Categories, ratios of the number of retracted articles from 1980–2010 to the number of 2010 WoS records vary from 0.00005 for History to 0.02034 for Anesthesiology (Fig. S1); indicating a higher retraction rate based on the size of the published literature in the latter category. This ratio also varies among subdisciplines within Medicine, from 0.00021 for Substance Abuse to 0.02034 for Anesthesiology (Fig. S1).

**Figure 2:**
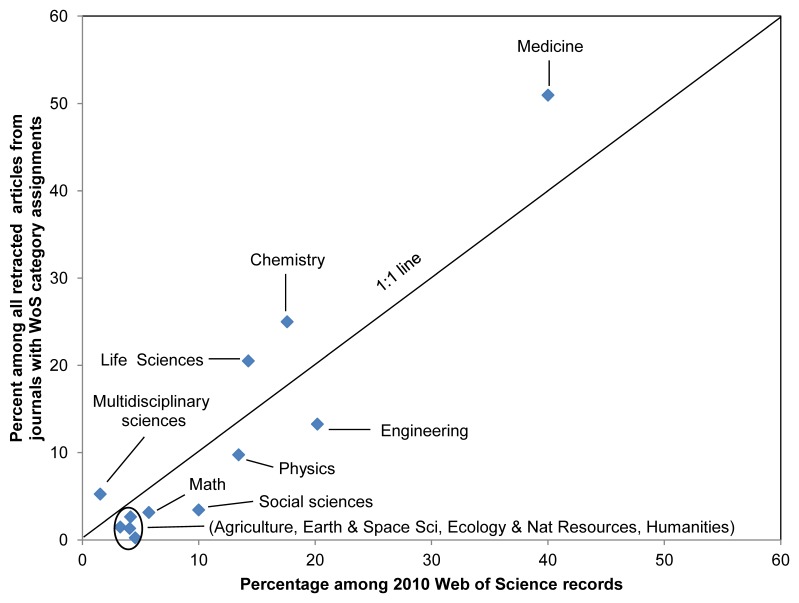
Percentage of retractions vs. percentage of 2010 Web of Science records among 12 broad scholarly fields. Field assignments were based on Web of Science Categories for 1,522 of the 1,796 journals with at least one retraction. The resulting 201 WoS Categories with at least one retracted article were then combined into these 12 broad subjects. Linear regression with intercept set to 0 yields: y = 1.1029x; R^2^ = 0.864.

The percentages of all retracted articles which are in the broad fields of Medicine, Chemistry, Life Sciences and Multidisciplinary Sciences are higher than the percentages of articles in these fields among all 2010 WoS records (Fig. 2). In contrast, percentages of all retracted articles in Engineering & Technology, Social Sciences, Mathematics, Physics, Agriculture, Earth & Space Sciences, Ecology & Natural Resources and Humanities are lower than their WoS percentages (Fig. 2). These two observations suggest that the former 4 fields have higher retraction rates, based on size of the published literature in each field, than the latter 8 fields.

### Justifications for Article Retraction

**Figure 3:**
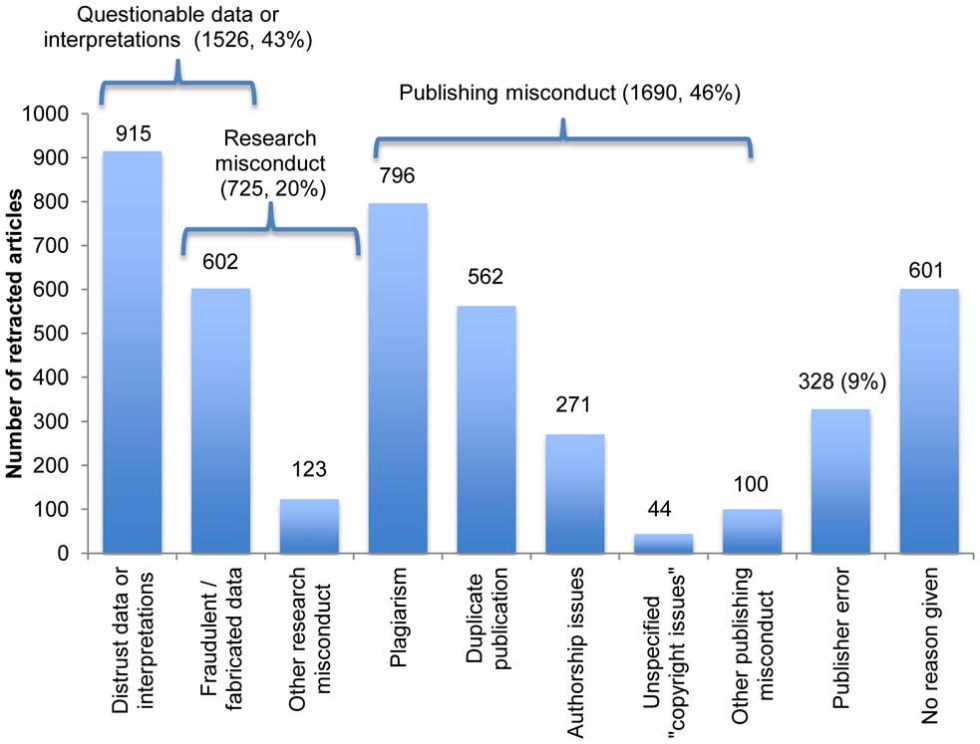
Justifications for retraction stated in the notices consulted, which accounted for 4,232 retracted articles. Only 20% of articles were retracted due to research misconduct, while more than twice that many were retracted due to publishing misconduct. Note that 42% were retracted because of “questionable data or interpretations.” Percentages are based on the 3,631 ( = 4,232−601) notices which stated the justification.

The retraction notices for 4,232 publications were obtained. Of these, the notices for 601 (14%) of them did not state why the publication was retracted. For the remaining 3,631, the counts of articles fitting each justification category in Table 2 are shown in Fig. 3. Alleged research misconduct was mentioned as a motivation for retraction of 20% of these articles; while 42% were motivated by questionable data or interpretations–whether due to alleged fraud, legitimate artifacts, unexplained irreproducibility, or re-interpretation of conclusions in the light of new facts. Publishing misconduct, primarily plagiarism and author-initiated duplicate publication, accounted for 47% of the retractions. All forms of publisher error represented 9%. These percentages add up to over 100% because some notices gave more than one justification for the retraction of one article.

### Proliferation of Retractions

**Figure 4:**
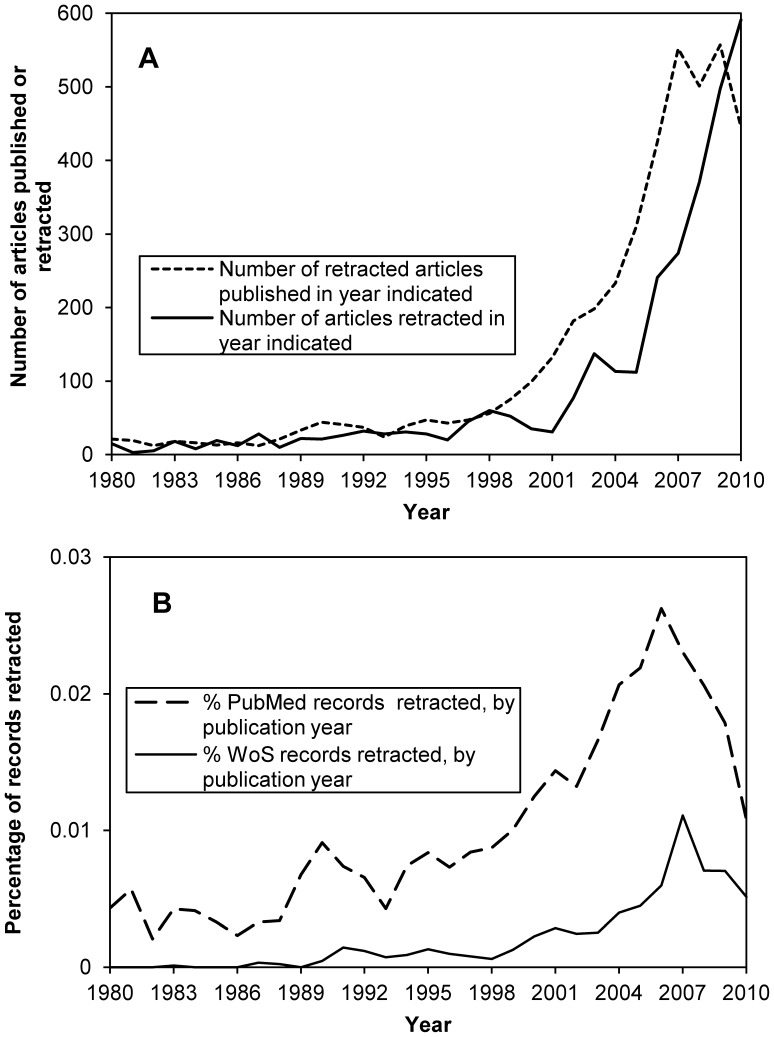
Yearly distribution of articles retracted from 1980–2010. (A) Counts for total number of articles published (n = 4,268) or retracted (n = 2,961) for each year from 1980–2010 from dataset of 4,449 retracted articles. These values differ because an additional 508 articles have been retracted thus far in 2011, and many online-only retraction notices do not indicate retraction year. (B) Percentages of records retracted are based on numbers of records with “retracted publication” in the document type field of PubMed or “retracted article” in the title field of Web of Science divided by total number of records for each year.

The year of retraction could be determined for 3,490 of the 4,449 retracted articles. This information was lacking for many online-only retraction notices. Only 21 articles retracted prior to 1980 were identified. Among the 2,961 publications retracted during 1980–2010, modest growth from 1980–2000 was followed by a period of increasing numbers of retractions per year (Fig. 4A). In 2010, 591 articles were retracted, an increase over the number retracted in 2001 by a factor of 19.06 (Fig. 4A). An additional 508 articles were retracted in 2011 (as of 22 Sep 2011), suggesting the total 2011 figure may exceed that for 2010. The dataset includes 780 publications (17.5% of all retractions) which were retracted while “in press,” but after being posted on the publisher’s website.

The percentage of all database entries (including articles, letters to the editor, etc.) for a given publication year which have been retracted (as of 17 Jul 2011) peaks at 0.0262% for 2006 in PubMed and 0.0111% for 2007 in WoS (Fig. 4B). Note that from 2001 to these peak years the percentage increases are only by a factor of 1.82 for PubMed and 3.82 for WoS (Fig. 4B) in contrast to the increase by a factor of 19.06 over the same period in Fig. 4A.

### The “repeat offenders”

The number of retracted articles authored by individual researchers in this survey ranged from 1 to 88. Of the 15 researchers with the largest number of retracted articles (Table 4), 9 had more than 20 retractions each, and 13 accounted for 391 (54%) of the global total of 725 retractions due to alleged research misconduct. The large numbers of retractions from these individuals skew overall data for individual years, countries, disciplines, and journals. For example, the ratio 2010 of retraction counts to 2001 retraction counts decreases from 19.06 to 15.94 if the retractions from repeat offenders are excluded from both years (Table 5).

**Table 4:** The top “repeat offenders” are collectively responsible for 52% of the world’s retractions due to alleged research misconduct.

Researcher	Retraction years	Country	Field of study	Number ofretractions	Justification givenfor retractions
Joachim Boldt^1^	2010–2011	Germany	Anesthesiology	88	Lack of IRB approval
Adrian Maxim^2^	2007	USA	Electrical engineering	48	Alleged data fraud andfictitious co-authors
H. Zhong^3^	2010	China	Chemistry	43	Alleged data fraud
Jon Hendrick Schön^4^	2002–2004	USA	Physics	33	Alleged data fraud
T. Liu^3^	2010	China	Chemistry	29	Alleged data fraud
Robert A. Slutsky^4^	1985–1987	USA	Cardiology	25	Alleged data fraud
Scott S. Reuben^4^	2009–2010	USA	Anesthesiology	24	Alleged data fraud
Naoki Mori^5^	2010–2011	Japan	Oncology	23	Alleged data fraud
Friedhelm Herrmann^6^	1997–2003	Germany	Oncology	22	Alleged data fraud
John R. Darsee^4^	1982–1984	USA	Cardiology	19	Alleged data fraud
Pattium Chiranjeevi^7^	2008	India	Chemistry	19	Plagiarism
Wataru Matsuyama^5^	2007–2010	Japan	Immunology	17	Alleged data fraud
Suresh Radhakrishnan^8^	2010	USA	Immunology	15	Alleged data fraud
M. Quik, G. Goldstein and collaborators	1993–1994	Canada	Physiology	15	Artifact (contamination)
Jon Sudbø^9^	2006–2007	Finland	Oncology	14	Alleged data fraud

These cases distort figures for individual journals, years, countries and subdisciplines, and are distributed throughout North America, Europe and Asia. Nine of the 15 are in medical fields.

1 Excluding one 2010 retraction, the Boldt case accounts for 87 (49%) of the 176 retractions for the entire EU-27 thus far in 2011.

2 According to the IEEExplore database, this author has allegedly fabricated data in 39 publications and co-authors of 14 additional publications.

3 The 72 retractions of these two authors represent 34% of China’s 210 retractions for 2010 and 8.9% of all 811 retractions for China.

4 These four authors account for 101 (7.5%) of all 1,355 USA retractions. It is noteworthy that Dr. Schön’s retractions include 10 articles from *Science* and 7 from *Nature*.

5 These two authors account for 40 (16%) of all 263 retractions for Japan.

7 This author accounts for 19 (6.8%) of all 280 retractions for India. Despite only 19 retractions, an institutional review alleged “plagiarizing and/or falsifying more than 70 research papers” [34] by this author.

1–6,8–9, Including 39 of Dr. Maxim’s articles with allegedly fabricated data, these 13 authors account for 391 (54%) of the world total of 725 retractions due to alleged research misconduct.

**Table 5:** Repeat offenders and “publication inflation” have both contributed to the rising number of retractions over the past 10 years.

Year	Total retractions	Total minus repeat offenders (TMRO)	WoS single year (SY)	TMRO/SY (×10^5^)	4-year intervals	WoS 4-year sum (4Y)	TMRO/4Y (×10^5^)
1998	–	–	1161571	–	–	–	–
1999	–	–	1187509	–	–	–	–
2000	–	–	1204571	–	–	–	–
**2001**	**31**	**31**	**1186681**	**2.61**	**1998–2001**	**4740332**	**0.65**
2002	77	63	1232725	5.03	1999–2002	4811486	1.29
2003	137	117	1267447	9.15	2000–2003	4891424	2.37
2004	112	111	1351993	8.28	2001–2004	5038846	2.22
2005	112	112	1426241	7.71	2002–2005	5278406	2.08
2006	238	232	1489702	15.77	2003–2006	5535383	4.25
2007	270	263	1562188	16.84	2004–2007	5830124	4.51
2008	369	337	1629820	20.49	2005–2008	6107951	5.47
2009	495	474	1682446	28.23	2006–2009	6364156	7.46
**2010**	**591**	**497**	**1665220**	**29.67**	**2007–2010**	**6539674**	**7.55**
**Ratio** 1	**19.06**	**15.94**	**1.40**	**11.36**	–	**1.38**	**11.55**

The 4-year sums of WoS records represent the time frame in which most (78.6%) of the articles that are retracted in any given year were published. Thus, changes in these sums represent changes in the size of the literature involved in most of that year’s retractions.

1 Ratio is the ratio of the 2010 value to the 2001 value for each column.

### “Publication inflation” and the Cumulative Growth of the Literature

Annual numbers of retractions have been increasing dramatically since 2001 (Fig. 4A, solid line). The effect of the growth of the published literature during the 10-year period from 2001–2010 is considered by comparing figures for these two individual years. Queries performed 17 Jul 2011 yielded ratios of total entries for 2010/2001 of 1.4 for WoS and 1.7 for PubMed. If counts for Total retractions Minus Repeat Offenders (TMRO) are adjusted for growth in the number of WoS records, the ratio of values for 2010/2001 decreases from 15.94 to 11.36 (Table 5). Since most retractions occur within 4 years of publication (Table S2), we also considered the change in ratios of TMRO for 2001 and 2010 divided by the size of the WoS literature for (1998–2001) and (2007–2010), respectively. However, we found little difference between the ratios of (2007–2010)/(1998–2001) and 2010/2001 for either WoS record counts (1.38 vs. 1.40) or WoS count-adjusted TMRO values (11.55 vs. 11.36, Table 5).

### Country Count Trends

**Figure 5:**
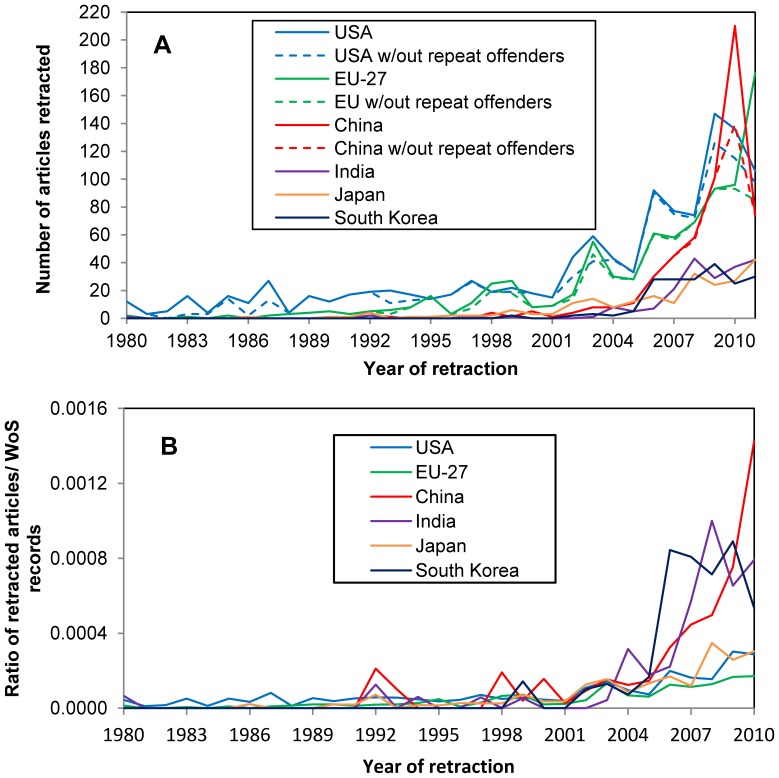
Yearly distribution of retracted articles based on author affiliation countries. (A) Number of articles retracted per year for EU-27, and top 5 non-EU countries. Note that China’s large spike in 2010 was mainly due to H. Zhong and T. Liu; and the EU-27 spike in 2011 is due to J. Boldt. Thus far in 2011, values for China (either red line), and EU-27 minus J. Boldt (green, dashed line) remain well below the USA values (either blue line). (B) Ratio of number of retracted articles/number of Web of Science articles for each year and geographic region. Because many retracted articles are not in Web of Science, these values are not true proportions, but allow for country comparisons.

The yearly distribution of the retraction of articles by authors from the European Union (EU-27) and top 5 non-EU countries by total retraction count is shown in Fig. 5. The USA and EU-27 clearly accounted for most retractions prior to 2005. Thereafter, the numbers from the Asian countries, particularly China, began to increase dramatically (Fig. 5A). The dashed lines represent counts excluding the articles from the repeat offenders (Table 4). Excluding the retracted publications by H. Zhong and T. Liu, China’s 2010 peak (dashed red line) drops to the level of the USA for 2010 (dashed blue line). If we exclude Joachim Boldt’s 2011 retractions, the dramatic rise in EU-27 retractions for 2011 through 22 Sep 2011 disappears (green dashed line).

The ratio of retractions to total WoS entries for 1980–2010 for each country (and the EU-27) normalizes the values, allowing comparisons of retraction rates between countries. A larger proportion of articles from authors in China, India and South Korea are being retracted in recent years when compared to those from authors in the USA, EU-27 and Japan (Fig. 5B).

### Retraction Authorities and “editorial expressions of concern”

The retraction notices for 4,232 articles were consulted. Those for 722 (17.1%) of them did not mention the authority calling for the retraction. Of the remaining 3,510, over half (1,970, 56.1%) mentioned either some or all of the authors; and a similar number (2,088, 59.5%) explicitly mentioned either the publisher, “the journal” or editor(s) (Table S4). Information provided by an investigation at the authors’ home institute or employer was mentioned in 358 (10.2%) as part of the decision to retract. Interestingly, this group included 276 (38%) of all 725 retractions due to alleged research misconduct. In contrast, 129 mentioned investigations by non-institutional watchdog agencies, such as the US Department of Health and Human Services, Office of Research Integrity (ORI).

When editors feel evidence is sufficient to question published data, but not (yet) sufficient to retract an article, they may issue an “editorial expression of concern” notice. As of 2 Aug 2011, such notices were found for 58 different articles, with 40 (70%) published since 2008 (Fig. S2). Although retraction usually occurs a few years post-publication ([6] and Table S2), roughly 1/3 (19/58) of the articles mentioned in these notices had been formally retracted by 2 Aug 2011. Like retraction notices [1], “expression of concern” notices often appear in high-impact journals, with 24/58 (40%) in *BMJ*, *Lancet*, *N Engl J Med*, *PNAS* or *Science*.

### Citation of Retracted Articles

As of 7 Jun 2012, the 1,837 items marked with “retracted article” in the title in WoS were collectively cited 41,562 times, including 4,311 times in 2010 alone. Retracted in 2009, the most highly-cited article [31], had 740 total WoS citations, including 187 for 2009–2012.

## Discussion

### Central Role of “repeat offenders”

Steen [2] termed authors with multiple retractions “repeat offenders.” His survey of 742 retractions (from PubMed, 2000–2010) yielded 7 authors with 5 or more retractions, and the top 2 authors with a combined total of 32 retracted articles [2]. This study, which included all scholarly fields, found 9 authors with 20 or more retractions each, and 434 retractions among the top 15 authors/groups (Table 4). These few aberrant cases are clearly “outliers” in a scientific community of millions. The repeat offenders identified in this study are globally distributed, with 7 from North America, 3 from Europe and 5 from Asia. Some authors have suggested that the stigma of misconduct should be decoupled from instances of article retractions [12], and the case of M. Quik, G. Goldstein and collaborators provides a good example. They discovered a potentially result-altering contaminant in their chemical standards after 15 articles had been published, and promptly sent retraction letters to the journals involved, *e.g.* [32].

The most extreme “repeat offender” cases appear frequently in editorials, *e.g.* [33]–[37]; and the data presented here provides a broader perspective in which their actions should be considered. The “soul-searching” in recent anesthesiology editorials on the Reuben and Boldt cases, *e.g.* [35], [36], [38] is reminiscent of cardiology editorials from the 1980s on the Slutsky and Darsee cases, *e.g.* [39], [40]. The Reuben and Boldt cases prompted a special 30-page editorial section on misconduct in the Mar 2011 issue of *Anesthesiology* [36]; in which one author asked “whether there is a specialty-related propensity for anesthesiologists to … commit academic fraud, or … [are Reuben and Boldt] … merely a statistical blip?” [38]. The data here strongly suggest the latter. Reuben and Boldt’s 98 articles retracted from strictly Anesthesiology journals accounted for 85% of all 115 articles that have ever been retracted from Anesthesiology journals. That only 17 articles had been retracted from all journals in this field prior to the Reuben case in 2009 argues strongly against rampant misconduct in anesthesiology. However, another dramatic case is currently unfolding in the field of anesthesiology, in which 193 published articles are at risk of being retracted [41].

The impact of repeat offenders on retraction counts for individual countries complicates direct cross-country comparisons. Their contributions among the top countries are: Schön, Slutsky, Reuben, and Darsee, 101 for the USA; Mori and Matsuyama, 40 for Japan; Chiranjeevi, 19 for India; Zhong and Liu, 72 for China; and Boldt and Hermann, 110 for Germany (Table 4). Subtracting their contributions blunts the retraction spikes for China in 2010 and the EU-27 in 2011 (Fig. 5A). A *Lancet* editorial used Zhong and Liu as examples to call on China to “reinvigorate standards for teaching research ethics” [42]. However, it could have cited the other repeat offenders to make equally dramatic cases for the inadequacy of standards in the USA, Japan, India, or Germany. These repeat offender cases should always be considered as the anomalies that they are, rather than held up as examples of the general state of research integrity.

The repeat offenders also dramatically skew results for individual journals (Zhong and Liu, *Acta Crystallogr E*, Table 3), subdisciplines (Boldt and Reuben, Anesthesiology; Zhong and Liu, Crystallography, Fig. S1) and years. Note that 10 of the top 15 cases have come to light since 2005 (Table 4), and the repeat offenders account for 15% of all 2010 retractions, and 24% of 2011 retractions catalogued thus far (Table 4).

### Rising Retraction Rates

While previous studies have documented the increasing number of retractions per year [1], [2], [5], [9]–[11], [19], [24]–[30], the inclusion of non-PubMed literature here yielded dramatically higher absolute numbers (Fig. 4). The growth of retracted non-PubMed literature in recent years (Fig. 1) underscores its importance in understanding the current impact of retracted literature on science as a whole, as well as non-medical scholarly fields of study. Despite the recent increases, retracted articles remain only fractions of a percent among all articles published in a given year or scholarly field. Some retracted articles undoubtedly eluded our search queries, so their actual number is higher than reported here. Queries of additional data sources, particularly those which thoroughly cover publications in narrow fields of study or from specific geographic regions, could reveal additional retracted articles. However, we are confident that a large proportion of articles which have been retracted were identified using all of the sources we consulted.

Various explanations for increasing numbers of retractions have been suggested. The repeat offenders and overall growth of the published literature are important contributors (Table 5); however, a dramatic rise in annual retraction counts in the past 10 years remains even after these factors are taken into account. Some debate on rising retraction rates focuses on whether “more cases are occurring” or simply “more cases are being caught” due to improved tools such as plagiarism-detecting software and the *Déjà vu* database [43] (http://spore.vbi.vt.edu/dejavu/browse/accessed 19 Mar 2012). Some journals now scan all submissions using CrossCheck, *e.g.,* [44]. Technological advances enable cut-and-paste plagiarism and en-mass multiple article submission. An outstanding case of the latter involved “… duplication of a paper that has already appeared in at least nine other publications” [45]! A companion article is in preparation which discusses in greater detail the likely motivations and possible steps which may reverse, or at least slow, the rising rate of retractions due to research and publishing misconduct.

Another contributor is the recent emergence of articles retracted while “in press” – *i.e.* those available to the research community on the publisher’s website, but retracted prior to volume, issue, and page assignment. This category, which did not exist 10 years ago, included 780 (17.5%) retractions in this survey. PubMed creates database records for such “in press” articles, while WoS does not. Thus records for hundreds of “in press” retracted articles exist in PubMed but not WoS (though this policy difference is not expected to affect the ratios in Fig. 4).

### Retractions Widespread among Disciplines

While the correlation between Impact Factor and number of retractions or “retraction index” [46] has been noted previously in the medical literature, this survey found retractions in 64% of all research journals with 2010 ISI Impact Factor of 9.000 or greater (Table S3). While some editors resist retracting articles, even when faced with overwhelming evidence of fraud, *e.g.,* [47], the fact remains that flawed research has slipped through the peer review process at most of the top journals in science and medicine. The use of WoS Categories allows objective disciplinary assignment of individual articles, though it is an imperfect system [48] due to topical mixing among the articles in a given journal. For example, the journal *Cell* is assigned only to the category of “Cell Biology,” though it contains individual articles that are relevant to Endocrinology, Oncology, Developmental Biology, etc.

The data generated in this study did not support the perception that research misconduct is primarily restricted to biomedical fields [49]. Large numbers of retracted articles, including those due to misconduct, are found outside of the medical literature (Figs. 1, 2). Chemistry and the Life Sciences, which overlap with Medicine in fields such as “Cell Biology” or “Chemistry, Medicinal,” exhibited disproportionately high retraction rates, similar to Medicine (Fig. 2). The higher proportion of database records marked as retracted in PubMed relative to WoS (Fig. 4) may reflect the lower retraction rates among the 8 other major disciplines in Fig. 2, which are all covered by WoS. However, many records in both databases fail to indicate the retracted status of articles. For example, some, *e.g.,* [22] are marked as retracted in WoS but not in PubMed (when checked on 7 Jun 2012). Thus, determining the true proportions of retracted articles in these databases would require surveying records for all articles known to be retracted.

### Full, Partial, Implicit, Explicit Retraction

Full retractions, *i.e.* retraction of the entire article, were called for in 4,120 (97%) of the 4,232 articles in our survey where the retraction notice was obtained. The remaining 112 (3%) cases retracted only a portion of an article. However, many notices labeled as “errata” report serious errors in published data–effectively “retracting” the original data or figure(s) without using the word “retract,” *e.g.,* [50]. On 7 Jun 2012, WoS included 295,957 records for errata, designated as publication type “correction” or “correction, addition,” with 12,189 for 2011 alone. The 112 “partial retractions” in our dataset could be considered “errata”.

The 9% of retractions due to publisher error (Fig. 3) is an underestimation. This study included only cases where “retraction notices” or “errata” explicitly call for the “retraction” or “withdrawal” of a publication. “Corrected and republished” articles typically involve severe flaws introduced by the publisher. However, many notices for them do not state the obvious implication that the original version is “retracted” and should not be consulted or cited. This also applies to many “duplicate publication” notices. In PubMed, 2,361 such records are indicated by “corrected and republished article” (n = 1,391, 7 Jun 2012) or “duplicate publication” (n = 973) in the publication type field. Of the flawed original articles among the 1,391 “corrected and republished” PubMed articles, only 7 were found on the list of 4,449 “retracted” publications in this study. Thus, thousands of these “implicit” retractions exist in addition to the 4,449 “explicit” retractions in the dataset used here.

### Retraction Authority

The retraction authority categories used here include all of the parties mentioned as guiding the decision to retract in all of the notices we obtained. There is some disagreement over the authority or responsibility of editors to declare an article as “retracted”. For example, one editor wrote: “Journals cannot retract–that is the obligation of authors … We can repudiate our association with a study.” [51]. Another suggests that allowing editors to take on the role of ethics police is “poison” to the scientific process [52]. Some editors retract articles without the authors’ consent, in what they perceive to be clear cases of fraud or error, due to a lack of response from authors, *e.g.,* [53]. This practice is supported by the Committee on Publication Ethics guidelines [54]. When co-authors disagree over retraction appropriateness, a “retraction of authorship” [55] or lack of consensus statement [56] may be published.

Many authors have stressed the responsibility of institutions and employers in fostering the responsible conduct of research (and publication) by their staff, *e.g.,* [57[-]58]. Their importance in facilitating retractions due to research misconduct is apparent, since the notices for 38% of articles retracted over allegations of research misconduct mentioned information provided by institute/employer investigations–far more than mentioned off-site watchdog organizations such as ORI (Table S4). However, obtaining clear-cut evidence of poor data integrity is often difficult for a variety of reasons [47], [59]–[60]. The “expression of concern” notice, *e.g.,* [61] rapidly alerts the research community to serious doubts about published data, particularly in the early stages of investigation or cases with ambiguous outcomes. Such notices may be subsequently “reaffirmed” [62] or “removed” [63] as appropriate. These notices are likely to become more common in the future given their relatively recent appearance and several-fold increase in number (Fig. S2) since a 2008 study on their characteristics was published [64].

### Citation of Retracted Articles

Efforts to inform researchers about retracted articles they may cite in the future have achieved only limited success [5]–[7]. However, the figures for citations of retracted articles may not be as alarming as they sound. Some citations do warn readers of an article’s retracted status [6]–[7], though ideally they all should. Since retraction does not automatically imply either “fraudulent data manipulation” or “questionable data” (Fig. 3), some authors defend the practice of citing valid data in retracted articles. For example, citing one of Schon’s retracted *Science* articles one author [65] noted: “This paper has been retracted … yet contains legitimate and innovating ideas that are now generally accepted.” In other cases, retraction can trigger a domino-effect, resulting in the retraction of subsequent articles with conclusions dependent on the retracted data or interpretations, *e.g.,* [66].

How can researchers stay informed of the constant march of retractions which may affect articles on which they rely for knowledge and cite? The role of publishers and bibliographic databases to properly mark retracted publications has been discussed [10]. The RetractionWatch (http://retractionwatch.wordpress.com) blog supplements the often minimal information retraction notices provide with that obtained directly from the authors, editors, or investigative committees involved. Passive online databases, such as the Retraction Database from Rutgers University (http://retract.rutgers.edu), are impractical as they require researchers to actively search for articles in their field. Solutions which do not require active and repetitive searching by researchers may be more effective. For example, CrossRef’s CrossMark initiative (http://www.crossref.org/crossmark/) is designed to help researchers identify the latest, definitive, “publisher-maintained” version available for an article of interest, as multiple versions of articles are typically generated during the publishing process or through incorporation of information from erratum or retraction notices. Another possibility could involve linking a truly comprehensive retraction database to the widely used reference management software tools, such as EndNote. This tool would scan a researchers’ personal EndNote library whenever opened, or when importing new references, and alert the user when a match between a known retracted article and a library entry is found.

### Conclusions

A very broad survey of the scholarly literature shows that retractions are widespread across disciplines and author affiliation countries; yet represent only small fractions of a percent among all publications for any given field, country, journal or year. While retracted articles and research integrity have received considerable attention in the medical literature, similar proportions of articles in the Life Sciences and Chemistry have also been retracted. Only limited proportions of articles have been retracted due to alleged research misconduct (20%) or loss of faith in the data or interpretations as published (43%). These low proportions support the call for de-coupling the stigma of “misconduct” from article retraction, and partially explain the continuing citation of retracted articles which may contain data believed to be valid. The effect of a limited number of prolific individuals on retraction counts for particular subsets can be very dramatic. These repeat offenders and overall growth of the published literature account for a substantial portion of the increase in the number of retractions over the past 10 years; though annual retraction figures adjusted for these factors have still increased dramatically. The central role of local authorities in investigating and providing evidence in cases of alleged misconduct was mentioned in a high proportion (38%) of such cases. Articles and editorials discussing article retraction and the separate, but related, issue of research integrity, should always consider them in these broad contexts.

## Supporting Information

Figure S1 **Ratios of retractions/WoS 2010 record counts for the 244 WoS Categories.** Because the published literature of scholarly fields differ in size, comparing retraction figures among different fields requires that they be normalized. Here we use the number of Web of Science records for 2010 as a measure of the size of the literature in each of the 244 WoS categories. The ratios of retractions/WoS record counts give some indication of relative proportions of articles in each field which have been retracted.
(XLSX)

Figure S2 **Yearly distribution of 58 “editorial expression of concern” notices from 30 different journals.** Such notices are expressions of opinion when ambiguity prevents outright retraction or in the early stages of investigating cases of questionable data.
(TIFF)

Table S1 Number of retracted articles included in previous large-scale studies of retraction attributes. Many narrowly-focused studies, e.g., [57] were excluded from this list.
(DOCX)

Table S2 Research journals with 2010 ISI Impact Factor of 9.000 or higher which have retracted articles. The 59/92 (64%) journals with retracted articles in this survey are indicated by asterisks (*).
(DOCX)

Table S3 Distribution of “Year of publication” among articles retracted since 1980.
(DOCX)

Table S4 Authorities specifically mentioned in retraction notices as being involved in the decision to retract, with percentage of the 3,510 articles for which the notice specified any authorities (except as noted).
(DOCX)

## References

[1] Cokol M, Iossifov I, Rodriguez-Esteban R, Rzhetsky A (2007) How many scientific papers should be retracted? EMBO Rep 8: 422–423. 10.1038/sj.embor.7400970 PMC1866214 17471252

[2] Steen RG (2011) Retractions in the medical literature: Who is responsible for scientific integrity? AMWA J 26: 2–8.

[3] Titus SL, Wells JA, Rhoades LJ (2008) Repairing research integrity. Nature 453: 980–982. 18563131 10.1038/453980a

[4] Fanelli D (2009) How many scientists fabricate and falsify research? A systematic review and meta-analysis of survey data. PLoS One 4: e5738. 19478950 10.1371/journal.pone.0005738 PMC2685008

[5] Budd JM, Sievert ME, Schultz TR, Scoville C (1999) Effects of article retraction on citation and practice in medicine. Bull Med Libr Assoc 87: 437–443. 10550028 PMC226618

[6] Korpela KM (2010) How long does it take for the scientific literature to purge itself of fraudulent material? The Breuning case revisited. Curr Med Res Opin 26: 843–847. 20136577 10.1185/03007991003603804

[7] Neale AV, Dailey RK, Abrams J (2010) Analysis of citations to biomedical articles affected by scientific misconduct. Sci Eng Ethics 16: 251–261. 19597966 10.1007/s11948-009-9151-4 PMC4141682

[8] Butler D, Hogan J (2007) Modellers seek reason for low retraction rates. Nature 447: 236–237. 10.1038/447236b 17507938

[9] Cokol M, Ozbay F, Rodriguez-Esteban R (2008) Retraction rates are on the rise. EMBO Rep 9: 2. 10.1038/sj.embor.7401143 PMC2246630 18174889

[10] Steen RG (2011) Retractions in the scientific literature: Is the incidence of research fraud increasing? J Med Ethics 37: 249–253. 21186208 10.1136/jme.2010.040923

[11] Nath SB, Marcus SC, Druss BG (2006) Retractions in the research literature: Misconduct or mistake? Med J Aust 185: 152–154. 16893357 10.5694/j.1326-5377.2006.tb00504.x

[12] Van Noorden R (2011) The trouble with retractions. Nature 478: 26–28. 21979026 10.1038/478026a

[13] Smith R (2003) When to retract? Reserve retraction for fraud and major error. BMJ 327: 883–884. 14563721 10.1136/bmj.327.7420.883 PMC218804

[14] Casati R (2010) On publishing. Soc Epistemol 24: 191–200.

[15] Errami M, Garner H (2008) A tale of two citations. Nature 451: 297–299. 18216832 10.1038/451397a

[16] Ehara S (2009) Changing environment against duplicate publications. Jpn J Radiol 27: 2–3. 19373525 10.1007/s11604-008-0282-3

[17] Xin H (2009) Retractions put spotlight on China’s part-time professor system. Science 323: 1280–1281. 19264995 10.1126/science.323.5919.1280

[18] Lee CS, Schrank A (2010) Incubating innovation or cultivating corruption? The developmental state and the life sciences in Asia. Soc Forces 88: 1231–1256.

[19] Woolley KL, Lew S, Stretton JA, Ely NJ, Bramich JR, et al (2011) Lack of involvement of medical writers and the pharmaceutical industry in publications retracted for misconduct: A systematic, controlled, retrospective study. Curr Med Res Opin 27: 1175–1182. 21473670 10.1185/03007995.2011.573546

[20] Ghazinoori S, Ghazinoori S, Azadegan-Mehr M (2011) Iranian academia. Evolution after revolution and plagiarism as a disorder. Sci Eng Ethics 17: 213–216. 20512425 10.1007/s11948-010-9206-6

[21] Wright K, McDaid C (2011) Reporting of article retractions in bibliographic databases and online journals. J Med Libr Assoc 99: 164–167. 21464856 10.3163/1536-5050.99.2.010 PMC3066576

[22] Davis AJ, Im YJ, Dubin JS, Tomer KB, Boss WF (2007) Arabidopsis phosphatidylinositol phosphate kinase 1 binds F-actin and recruits phosphatidylinositol 4-kinase b1 to the actin cytoskeleton. J Biol Chem 282: 14121–14131 (retracted in: Anon (2009) Corrections. J Biol Chem 284: 16060).. 17379598 10.1074/jbc.M611728200

[23] Bosch X (2011) Treat ghostwriting as misconduct. Nature 469: 472. 10.1038/469472c 21270878

[24] Snodgrass GL, Pfeifer MP (1992) The characteristics of medical retraction notices. Bull Med Libr Assoc 80: 328–334. 1422502 PMC225694

[25] Budd JM, Sievert M, Schultz TR (1998) Phenomena of retractions: Reasons for retraction and citations to the publications. JAMA 280: 296–297. 9676689 10.1001/jama.280.3.296

[26] Foo JYA (2011) A retrospective analysis of the trend of retracted publications in the field of biomedical and life sciences. Sci Eng Ethics 17: 459–468. 20517712 10.1007/s11948-010-9212-8

[27] Wager E, Williams P (2011) Why and how do journals retract articles? An analysis of Medline retractions 1988–2008. J Med Ethics 37: 567–570. 21486985 10.1136/jme.2010.040964

[28] Redman BK, Yarandi HN, Merz JF (2008) Empirical developments in retraction. J Med Ethics 34, 807–809. 10.1136/jme.2007.023069 18974415

[29] Steen RG (2011) Retractions in the medical literature: How many patients are put at risk by flawed research? J Med Ethics 37: 688–692. 21586404 10.1136/jme.2011.043133

[30] Steen RG (2011) Retractions in the scientific literature: Do authors deliberately commit research fraud? J Med Ethics 37: 113–117. 21081306 10.1136/jme.2010.038125

[31] Reyes M, Lund T, Lenvik T, Aguiar D, Koodie L, et al (2001) Purification and ex vivo expansion of postnatal human marrow mesodermal progenitor cells. Blood 98: 2615–2625. 11675329 10.1182/blood.v98.9.2615

[32] Quik M, Afar R, Geertsen S, El-Bizri H, Trifaro JM, et al (1993) Retraction letter. Mol Pharmacol 44: 680. 8371721

[33] Schulz WG (2008) A massive case of fraud. Chem Eng News 86: 37–38.

[34] Service RF (2008) Chemist found responsible for ethical breaches. Science 319: 1170–1171. 10.1126/science.319.5867.1170b 18309053

[35] Shafer SL (2009) Tattered threads. Anesthes Analges 108: 1361–1363. 10.1213/ane.0b013e3181a16846 19372305

[36] Shafer SL (2011) You will be caught. Anesthes Analges 112: 491–493. 10.1213/ANE.0b013e3182095c73 21350222

[37] Anon (2011) They did a bad bad thing. Nat Chem 3: 337. 21505484 10.1038/nchem.1042

[38] Mutch WAC (2011) Academic fraud: Perspectives from a lifelong anesthesia researcher. Can J Anesth 58: 782–788. 21643872 10.1007/s12630-011-9523-5

[39] Relman AS (1983) Lessons from the Darsee affair. N Engl J Med 308: 1415–1417. 6843634 10.1056/NEJM198306093082311

[40] Engler RL, Covell JW, Friedman PJ, Kitcher PS, Peters RM (1987) Misrepresentation and responsibility in medical research. N Engl J Med 317: 1383–1389. 3317039 10.1056/NEJM198711263172205

[41] Rasmussen LS, Yentis SM, Gibbs N, Kawamoto M, Shafer SL, et al. (2012) Joint Editors-in-Chief request for determination regarding papers published by Dr. Yoshitaka Fujii. 23 pp. Available: http://www.springer.com/cda/content/document/cda_downloaddocument/Fujii_Joint_Editorial_Request_Regarding_Dr_Yoshitaka_Fujii.pdf.

[42] Anon (2010) Scientific fraud: Action needed in China. Lancet 375: 94. 10.1016/S0140-6736(10)60030-X 20109871

[43] Errami M, Sun Z, Long TC, George AC, Garner HR (2009) Déjà vu: A database of highly similar citations in the scientific literature. Nucl Acids Res 37: D921–D924. 18757888 10.1093/nar/gkn546 PMC2686470

[44] Kleinert S (2011) Checking for plagiarism, duplicate publication, and text recycling. Lancet 377: 281–282. 10.1016/S0140-6736(11)60565-5 21515152

[45] Anon (2007) Retraction notice to: “Properties of organic light-emitting diodes by aluminum cathodes modification using Ar+ ion beam” [Org. Electron. 6 (4) (2005) 149–160]. Org Electron 8: iii.

[46] Fang FC, Casadevall A (2011) Retracted science and retraction index. Infect Immun 79: 3855–3859. 21825063 10.1128/IAI.05661-11 PMC3187237

[47] Jureidini JN, McHenry L (2011) Conflicted medical journals and the failure of trust. Accountabil Res 18: 45–54. 10.1080/08989621.2011.542683 21287414

[48] Leydesdorff L, Rafols I (2009) A global map of science based on the ISI subject categories. J Am Soc Info Sci Tech 60: 348–362.

[49] Dyer C (2011) The fraud squad. BMJ 342: d4017. 21712330 10.1136/bmj.d4017

[50] Anon (2000) Corrections. Proc Natl Acad Sci USA 97: 3782.10836971

[51] DXF (1988) Editor’s comment and action. Arch Gen Psychiat 45: 686.

[52] Munk-Jorgensen P (2010) Authors are not criminals and editors should not be policemen. Epidemiol Psichiat Soc 19: 193–195. 21261211

[53] Anon (2007) Retraction: Reduced semen quality in chronic prostatitis patients that induce the release of apoptotic protein Omi/HtrA2 from spermatozoa. Prostate Cancer Prostat Dis 10: 398. 10.1038/sj.pcan.4501016 18049464

[54] Wager E, Barbour W, Yentis S, Kleinert S (2009) Retractions: Guidance from the Committee on Publication Ethics (COPE). Available: http://publicationethics.org/files/retraction%20guidelines.pdf. 10.1111/j.1467-789X.2009.00702.x 20653849

[55] Anon (2010) Retraction of authorship. Urol Int 84: 484.

[56] Elsheikh E, Uzunel M, Nowak G (2011) Retraction. Blood 117: 6740.21546458

[57] Sox HC, Rennie D (2006) Research misconduct, retraction, and cleansing the medical literature. Lessons from the Poehlman case. Ann Intern Med 144: 609–613. 16522625 10.7326/0003-4819-144-8-200604180-00123

[58] Gillman MA (2011) Checking for plagiarism, duplicate publication, and text recycling. Lancet 377: 1403. 10.1016/S0140-6736(11)60566-7 21515151

[59] Smith J, Godlee F (2005) Investigating allegations of scientific misconduct. Journals can do only so much; institutions need to be willing to investigate. BMJ 331: 245–246. 16051990 10.1136/bmj.331.7511.245 PMC1181252

[60] White C (2005) Suspected research fraud. Difficulties of getting at the truth. BMJ 331: 281–288. 16052022 10.1136/bmj.331.7511.281 PMC1181273

[61] Anon (2010) Editor’s note: Note of concern. Am J Pathol 177: 2147. 20884964 10.2353/ajpath.2010.100773 PMC2947308

[62] Curfman GD, Morrissey S, Drazen JM (2006) Expression of concern reaffirmed. N Engl J Med 354: 1193. 16495386 10.1056/NEJMe068054

[63] Drazen JM, Ingelfinger JR, Curfman GD (2003) Removal of expression of concern. N Engl J Med 349: 1965. 14614172 10.1056/NEJMe038188

[64] Noonan B, Parrish D (2008) Expressions of concern and their uses. Learned Publishing 21: 209–213.

[65] Verlaak S, Cheyns D, Debucquoy M, Arkipov V, Heremans P (2004) Numerical simulation of tetracene light-emitting transistors. A detailed balance of exciton processes. Appl Phys Lett 85: 2405–2407.

[66] Anon (2008) Retraction. Immunol Rev 224: 295.18759935

