# Supplementary material for: A Comprehensive Survey of Retracted Articles from the Scholarly Literature
Source: PLoS One. 2012 Oct 24;7(10):e44118. doi: 10.1371/journal.pone.0044118 (PMC3480361; doi:10.1371/journal.pone.0044118)
Supplement: Table S1 — Number of retracted articles included in previous large-scale studies of retraction attributes. Many narrowly-focused studies, e.g., [57] were excluded from this list. (DOCX) [file pone.0044118.s003.docx]

**Supplementary Table S1. Number of retracted articles included in previous large-scale studies of retraction attributes.** Many narrowly-focused studies, e.g., [57] were excluded from this list.

| 97 from Medline (1975 to Aug 1991) [24] |
| --- |
| 235 from Medline (1966-1996) [5, 25] |
| 303 selected from 1295 retracted Medline entries [26] |
| 312 selected from 870 PubMed (1988-2008) [27] |
| 328 from PubMed (1995-2004) [28] |
| 395 from Medline (1982-2002) [11] |
| 520 selected from Medline (1966-2008 Feb 18) [19] |
| 596 from Medline (1950-2004) [1] |
| 788 from PubMed (2000-2010) [2, 10, 29-30] |
| 871 from Medline (as of 21 Oct 2007) [9] |
| **~3,600-4,300 (for each attribute) of 4,449 retracted articles – Current study** |
