# Supplementary material for: A Comprehensive Survey of Retracted Articles from the Scholarly Literature
Source: PLoS One. 2012 Oct 24;7(10):e44118. doi: 10.1371/journal.pone.0044118 (PMC3480361; doi:10.1371/journal.pone.0044118)
Supplement: Table S2 — Research journals with 2010 ISI Impact Factor of 9.000 or higher which have retracted articles. The 59/92 (64%) journals with retracted articles in this survey are indicated by asterisks (*). (DOCX) [file pone.0044118.s004.docx]

**Supplementary Table S2. Research journals with 2010 ISI Impact Factor of 9.000 or higher.** The 59/92 (64%) journals with retracted articles in this survey are indicated by asterisks (*).

| ACS Nano | EJC Suppl | Mol Syst Biol |
| --- | --- | --- |
| *Acta Crystallogr A | *EMBO J | Nano Lett |
| *Adv Mater | Energ Environ Sci | Nano Today |
| *Am J Hum Genet | Energy Educ Sci Tech | *Nat Biotechnol |
| *Am J Psychiat | Eur Cells Mater | *Nat Cell Biol |
| *Am J Resp Crit Care | Eur Heart J | Nat Chem |
| *Angew Chem Int Edit | *Gastroenterology | *Nat Chem Biol |
| *Ann Intern Med | *Gene Dev | *Nat Genet |
| *Ann Neurol | *Genome Res | *Nat Geosci |
| *Ann Rheum Dis | *Gut | *Nat Immunol |
| *Arch Gen Psychiat | *Hepatology | *Nat Mater |
| *Arch Intern Med | *Immunity | *Nat Med |
| Astrophys J Suppl S | *J Allergy Clin Immun | Nat Methods |
| Behav Brain Sci | *J Am Chem Soc | Nat Nanotechnol |
| *Blood | *J Am Coll Cardiol | Nat Neurosci |
| *Brain | *J Cell Biol | Nat Photonics |
| Brief Bioinform | *J Clin Invest | Nat Phys |
| *Brit Med J | *J Clin Oncol | *Nat Struct Mol Biol |
| *Can Med Assoc J | *J Exp Med | *Nature |
| Cancer Cell | *J Hepatol | *Neuron |
| *Cell | J Mol Cell Biol | *New Engl J Med |
| *Cell Death Differ | *J Natl Cancer I | *P Natl Acad Sci USA |
| Cell Host Microbe | *J Photoch Photobio C | *Plant Cell |
| Cell Metab | *JAMA | PLoS Biol |
| *Cell Res | *Lancet | *PLoS Genet |
| Cell Stem Cell | Lancet Infect Dis | PLoS Med |
| *Circ Res | Lancet Neurol | *PLoS Pathog |
| *Circulation | *Lancet Oncol | Psychol Bull |
| *Dev Cell | *Mol Aspects Med | *Science |
| Drug Resist Update | Mol Cell | Syst Biol |
| Ecol Lett | Mol Psychiatr |  |
