# Supplementary material for: A Comprehensive Survey of Retracted Articles from the Scholarly Literature
Source: PLoS One. 2012 Oct 24;7(10):e44118. doi: 10.1371/journal.pone.0044118 (PMC3480361; doi:10.1371/journal.pone.0044118)
Supplement: Table S3 — Distribution of “Year of publication” among articles retracted since 1980. (DOCX) [file pone.0044118.s005.docx]

**Supplementary Table S3.** Distribution of “Year of publication” among articles retracted since 1980.

| Year of publication | 72 | 73 | 74 | 75 | 76 | 77 | 78 | 79 | 80 | 81 | 82 | 83 | 84 | 85 | 86 | 87 | 88 | 89 | 90 | 91 | 92 | 93 | 94 | 95 | 96 | 97 | 98 | 99 | 00 | 01 | 02 | 03 | 04 | 05 | 06 | 07 | 08 | 09 | 10 | 11 | Total Retracts | Total minus repeat offenders |
| --- | --- | --- | --- | --- | --- | --- | --- | --- | --- | --- | --- | --- | --- | --- | --- | --- | --- | --- | --- | --- | --- | --- | --- | --- | --- | --- | --- | --- | --- | --- | --- | --- | --- | --- | --- | --- | --- | --- | --- | --- | --- | --- |
| Year of retraction |  |  |  |  |  |  |  |  |  |  |  |  |  |  |  |  |  |  |  |  |  |  |  |  |  |  |  |  |  |  |  |  |  |  |  |  |  |  |  |  |  |  |
| 1980 |  |  |  |  |  | 1 | 5 | 5 | 4 |  |  |  |  |  |  |  |  |  |  |  |  |  |  |  |  |  |  |  |  |  |  |  |  |  |  |  |  |  |  |  | 15 | 15 |
| 1981 |  |  |  |  | 1 |  |  |  | 1 | 1 |  |  |  |  |  |  |  |  |  |  |  |  |  |  |  |  |  |  |  |  |  |  |  |  |  |  |  |  |  |  | 3 | 3 |
| 1982 |  |  |  |  |  |  |  |  | 1 | 4 |  |  |  |  |  |  |  |  |  |  |  |  |  |  |  |  |  |  |  |  |  |  |  |  |  |  |  |  |  |  | 5 | 0 |
| 1983 |  |  |  |  |  |  | 1 | 2 | 7 | 5 |  | 3 |  |  |  |  |  |  |  |  |  |  |  |  |  |  |  |  |  |  |  |  |  |  |  |  |  |  |  |  | 18 | 5 |
| 1984 |  |  |  |  |  |  |  | 1 | 1 | 2 | 1 | 3 |  |  |  |  |  |  |  |  |  |  |  |  |  |  |  |  |  |  |  |  |  |  |  |  |  |  |  |  | 8 | 7 |
| 1985 |  |  |  |  |  |  | 1 | 3 |  | 4 | 2 | 4 | 2 | 3 |  |  |  |  |  |  |  |  |  |  |  |  |  |  |  |  |  |  |  |  |  |  |  |  |  |  | 19 | 17 |
| 1986 |  |  |  |  |  |  |  |  |  |  |  | 2 | 6 | 3 | 1 |  |  |  |  |  |  |  |  |  |  |  |  |  |  |  |  |  |  |  |  |  |  |  |  |  | 12 | 3 |
| 1987 |  |  |  |  |  |  |  | 1 | 3 | 1 | 4 | 3 | 5 | 3 | 7 | 1 |  |  |  |  |  |  |  |  |  |  |  |  |  |  |  |  |  |  |  |  |  |  |  |  | 28 | 14 |
| 1988 |  |  |  |  |  |  |  |  | 2 | 1 | 2 | 1 |  |  |  | 2 | 2 |  |  |  |  |  |  |  |  |  |  |  |  |  |  |  |  |  |  |  |  |  |  |  | 10 | 10 |
| 1989 | 1 |  |  |  |  | 1 | 1 |  |  |  | 2 | 1 |  | 1 | 4 | 1 | 3 | 5 |  |  |  |  |  |  |  |  |  |  |  |  |  |  |  |  |  |  |  |  |  |  | 20 | 20 |
| 1990 |  |  |  |  |  |  |  |  |  |  |  |  |  | 1 |  | 2 | 5 | 9 | 4 |  |  |  |  |  |  |  |  |  |  |  |  |  |  |  |  |  |  |  |  |  | 21 | 21 |
| 1991 |  |  |  |  |  |  |  |  |  |  |  |  | 1 | 1 | 1 |  | 3 | 5 | 10 | 5 |  |  |  |  |  |  |  |  |  |  |  |  |  |  |  |  |  |  |  |  | 26 | 26 |
| 1992 |  |  |  |  |  |  |  |  |  |  |  |  | 1 |  |  | 1 |  | 2 | 11 | 16 | 1 |  |  |  |  |  |  |  |  |  |  |  |  |  |  |  |  |  |  |  | 32 | 32 |
| 1993 |  |  |  |  |  |  |  |  |  |  |  |  |  |  | 2 | 2 |  | 2 | 5 | 7 | 10 |  |  |  |  |  |  |  |  |  |  |  |  |  |  |  |  |  |  |  | 28 | 17 |
| 1994 |  |  |  |  |  |  | 1 |  | 1 |  | 1 |  | 1 |  |  | 1 |  | 1 | 5 | 3 | 8 | 5 | 4 |  |  |  |  |  |  |  |  |  |  |  |  |  |  |  |  |  | 31 | 27 |
| 1995 |  |  |  |  |  |  |  |  |  |  |  |  |  |  |  |  |  | 1 | 1 | 1 | 3 | 1 | 13 | 8 |  |  |  |  |  |  |  |  |  |  |  |  |  |  |  |  | 28 | 28 |
| 1996 |  |  |  |  |  |  |  |  |  |  |  |  |  |  |  | 1 |  | 1 |  | 1 |  | 4 | 2 | 8 | 3 |  |  |  |  |  |  |  |  |  |  |  |  |  |  |  | 20 | 20 |
| 1997 |  |  |  |  |  |  |  |  |  |  |  | 1 |  |  |  |  | 1 |  | 3 | 1 | 4 | 1 | 5 | 12 | 13 | 4 |  |  |  |  |  |  |  |  |  |  |  |  |  |  | 45 | 41 |
| 1998 |  |  |  |  |  |  |  |  |  |  |  |  |  |  | 1 | 1 | 2 | 2 | 2 | 2 |  | 5 | 10 | 8 | 8 | 15 | 4 |  |  |  |  |  |  |  |  |  |  |  |  |  | 60 | 55 |
| 1999 |  |  |  |  |  | 1 |  |  |  |  |  |  |  |  |  |  | 1 | 2 |  | 1 | 5 |  | 3 | 3 | 9 | 10 | 11 | 6 |  |  |  |  |  |  |  |  |  |  |  |  | 52 | 43 |
| 2000 |  |  |  |  |  |  |  |  |  |  |  |  |  |  |  |  |  | 1 | 1 |  |  | 2 |  |  | 1 | 3 | 6 | 13 | 8 |  |  |  |  |  |  |  |  |  |  |  | 35 | 35 |
| **2001** |  |  |  |  |  |  |  |  |  |  |  |  |  |  |  |  |  |  | **2** | **2** | **1** |  |  | **2** | **1** | **1** | **2** | **6** | **8** | **6** |  |  |  |  |  |  |  |  |  |  | **31** | **31** |
| 2002 |  |  |  |  |  |  |  |  |  |  |  |  |  |  |  |  |  |  |  |  |  |  |  |  |  |  | 3 | 4 | 21 | 36 | 13 |  |  |  |  |  |  |  |  |  | 77 | 63 |
| 2003 |  |  |  |  |  |  |  |  |  |  |  |  |  |  |  |  | 3 | 1 |  | 1 | 1 | 2 | 1 | 1 |  | 3 | 2 | 5 | 14 | 18 | 65 | 20 |  |  |  |  |  |  |  |  | 137 | 117 |
| 2004 |  |  |  |  |  |  |  |  |  |  |  |  |  |  |  |  |  |  |  |  |  |  |  | 1 |  | 1 | 7 | 6 | 3 | 8 | 11 | 50 | 25 |  |  |  |  |  |  |  | 112 | 111 |
| 2005 |  |  |  |  |  |  |  |  | 1 | 1 |  |  |  |  |  |  |  |  |  |  | 1 |  | 1 |  |  | 2 | 3 | 3 | 2 | 7 | 5 | 15 | 39 | 32 |  |  |  |  |  |  | 112 | 112 |
| 2006 |  |  |  |  |  |  |  |  |  |  |  |  |  |  |  |  |  |  |  |  |  |  |  | 1 |  | 3 | 3 | 1 | 5 | 7 | 17 | 18 | 26 | 62 | 95 |  |  |  |  |  | 238 | 232 |
| 2007 |  |  |  |  |  |  |  |  |  |  |  |  |  |  |  |  |  |  |  | 1 |  | 1 |  |  |  | 1 | 2 |  | 3 | 4 | 10 | 15 | 18 | 38 | 86 | 91 |  |  |  |  | 270 | 263 |
| 2008 |  |  |  |  |  |  |  |  |  |  |  |  |  |  |  |  |  |  |  |  |  |  |  |  |  |  | 4 | 6 | 4 | 11 | 7 | 9 | 24 | 31 | 60 | 108 | 105 |  |  |  | 369 | 337 |
| 2009 |  |  | 1 |  |  |  |  |  |  |  |  |  |  |  |  |  |  |  |  |  | 1 |  |  |  | 2 | 2 | 3 | 7 | 9 | 7 | 10 | 16 | 20 | 31 | 38 | 58 | 137 | 153 |  |  | 495 | 474 |
| **2010** |  |  |  |  |  |  |  |  |  |  |  |  |  |  |  |  |  |  |  |  |  | **1** |  | **1** | **2** |  | **2** | **5** | **3** | **3** | **13** | **7** | **20** | **27** | **29** | **119** | **63** | **142** | **154** |  | **591** | **497** |
| 2011 |  |  |  |  |  |  |  |  |  |  |  |  |  |  |  |  |  | 1 |  |  | 2 | 1 |  |  | 1 | 2 | 2 | 8 | 12 | 14 | 14 | 22 | 19 | 25 | 30 | 28 | 44 | 68 | 142 | 71 | 506 | 399 |
| Grand Total | 1 | 0 | 1 | 0 | 1 | 3 | 9 | 12 | 21 | 19 | 12 | 18 | 16 | 12 | 16 | 12 | 20 | 33 | 44 | 41 | 37 | 23 | 39 | 45 | 40 | 47 | 54 | 70 | 92 | 121 | 165 | 172 | 191 | 246 | 338 | 404 | 349 | 363 | 296 | 71 | 3454 | 3075 |
