# Supplementary material for: A Comprehensive Survey of Retracted Articles from the Scholarly Literature
Source: PLoS One. 2012 Oct 24;7(10):e44118. doi: 10.1371/journal.pone.0044118 (PMC3480361; doi:10.1371/journal.pone.0044118)
Supplement: Table S4 — Authorities specifically mentioned in retraction notices as being involved in the decision to retract, with percentage of the 3,510 articles for which the notice specified any authorities (except as noted). (DOCX) [file pone.0044118.s006.docx]

**Supplementary Table S4. Authorities specifically mentioned in retraction notices as being involved in the decision to retract, with percentage of the 3,510 articles for which the notice specified any authorities (except as noted).**

| **Retraction authority** | **Number and percentage** |
| --- | --- |
| Publisher or "journal" | 762 (21.7%) |
| Editor | 1,636 (46.6%) |
| Publisher, “journal” or editor | 2,088 (59.5%) |
| Some authors | 468 (13.3%) |
| All authors^1^ | 1,501 (42.8%) |
| All or some authors | 1,969 (56.1%) |
| Local investigative committee | 358 (10.2%) |
| ORI, NSF or FDA^2^ | 129 (3.7%) |
| Lawyer or ”court documents” | 23 (0.6%) |
| Unspecified | 722 (17.1%^3^) |

^1^ “All authors” were assumed when notices simply said “The authors wish to retract…”

^2^ ORI = U.S. Department of Health and Human Services, Office of Research Integrity; NSF = U.S. National Science Foundation; FDA = U.S. Food and Drug Administration

^3^ This is the percentage of all 4,232 articles for which retraction notices were consulted.
